# Activated AMP-activated protein kinase prevents hepatic steatosis, oxidative stress and inflammation in primary chicken hepatocytes

**DOI:** 10.3389/fphys.2022.974825

**Published:** 2022-09-08

**Authors:** Yao Yao, Longlong Li, Huihui Wang, Ying Yang, Haitian Ma

**Affiliations:** ^1^ Key Laboratory of Animal Physiology and Biochemistry, College of Veterinary Medicine, Nanjing Agricultural University, Nanjing, China; ^2^ MOE Joint International Research Laboratory of Animal Health and Food Safety, College of Veterinary Medicine, Nanjing Agricultural University, Nanjing, China

**Keywords:** fatty liver hemorrhagic syndrome, AMP-activated protein kinase signaling pathway, lipid metabolism disorders, oxidative stress, primary chicken hepatocytes

## Abstract

Fatty liver hemorrhagic syndrome (FLHS) in laying hens, a nutritional metabolic disorder disease, can lead to the decline of laying rate, shortening of laying peak period and increase of mortality, which seriously constrain the sustainable development of layer industry. Until now, there is no effective strategies can prevent and control the occurrence of fatty liver hemorrhagic syndrome in laying hens. The AMP-activated protein kinase (AMPK), a major sensor of cellular energy status, acts a crucial role in regulating lipid metabolism, oxidative stress and inflammatory responses in body. However, the potential molecular mechanisms about AMP-activated protein kinase signal in controlling the occurrence of fatty liver hemorrhagic syndrome are remain unclear. In present study, we found that the phosphorylated AMP-activated protein kinase (Thr172) protein level was markedly reduced in palmitic acid plus oleic acid (PO)-induced primary chicken hepatocytes. Moreover, blocked AMP-activated protein kinase signal by AMP-activated protein kinase inhibitor compound C obviously exacerbated lipid metabolism disorders, oxidative stress and inflammatory response triggered by palmitic acid plus oleic acid in primary chicken hepatocytes. Nevertheless, the lipid metabolism disorders, oxidative stress and inflammatory response challenged by palmitic acid plus oleic acid were obviously alleviated through activation of AMP-activated protein kinase signal with AMP-activated protein kinase activator AICAR in hepatocytes. In addition, we found that the beneficial effects of AMP-activated protein kinase signal in relieving lipid metabolism disorders, oxidative stress and inflammatory response are achieved by activating the nuclear factor erythroid 2-related factor 2 (NRF-2)/kelch-like ECH-associated protein 1 (KEAP1) pathway and inhibiting the NF-κB pathway in PO-stimulated primary chicken hepatocytes. Collectively, our data demonstrated that AMP-activated protein kinase acts as a potential target for the prevention of fatty liver hemorrhagic syndrome occurrence in laying hens.

## Introduction

Fatty liver hemorrhagic syndrome (FLHS), a nutritional metabolic disease, is mainly causes by the imbalance of dietary nutrients and the lack of trace elements, which can result in a large amount of lipid accumulation and liver dysfunction in laying hens. Usually, FLHS occurs in the peak period of laying or high-yield caged laying hens, and that is characterized by the hepatic steatosis, oxidative stress, inflammation and bleeding, and accompanied by the abnormal abdominal fat deposition and dyslipidemia. The occurrences of FLHS can lead to the decline of laying rate, the shortening of laying peak period and the increase of mortality, that arises a serious economic loss for the laying industry (Wolford and Polin, 1972; Trott et al., 2014). Previous study reported that FLHS is the main inducer for the death in laying hens, and that up to 74% mortality in caged laying hens is attribute to the occurrences of FLHS (Rozenboim et al., 2016). At present, with the popularity of intensive, large-scale and intelligent breeding methods in laying hens, the incidence rate of FLHS is increased year by year, and that had been paid the close attention by the layer farming industry. However, the strategies in actual production aimed to alleviate the occurrences of FLHS are still limited; therefore, it is still necessary to investigate the potential molecular mechanisms for contributing to prevent the progression of FLHS in laying hens.

AMP-activated protein kinase (AMPK) is the major sensor and regulation factor for cellular energy status, and which acts a crucial role in regulating lipid metabolism, glucose metabolism, oxidative stress and inflammatory responses in body (Green et al., 2022). Previous study found that the inhibition of AMPK signals is associated with the progression of nonalcoholic fatty liver disease (NAFLD) (Zhao and Saltiel, 2020). Moreover, liver-specific AMPK knockout mice exhibited more severe pathology characteristic of NAFLD, including enhanced hepatic steatosis, oxidative stress, liver function damage and inflammatory responses, which indicated that AMPK is a potential target for the treatment and prevention of NAFLD (Strzyz, 2020). Recently, our series studies had confirmed that activated AMPK can prevent the occurrences of NAFLD/NASH induced by high-fat/high-cholesterol diets in rodents or the lipid metabolism disorders, oxidative stress and inflammatory response challenged by palmitic acid/oleic acid stimulation in hepatocytes (Li et al., 2005; Li et al., 2021). Interestingly, both the FLHS in laying hens and the NAFLD in humans are characterized by lipid metabolism disorders and oxidative stress in the liver, and simultaneously accompanied by obesity and dyslipidemia. Therefore, AMPK may exert an important function in FLHS progression. Although previous study showed that *AMPKα1* mRNA levels were significantly reduced in the liver tissue of laying hens with FLHS (Gao et al., 2019), whether AMPK acts a crucial role in controlling the occurrence and development of FLHS still needs further exploration.

Hence, the present study aimed to investigate the beneficial roles and potential mechanisms of AMPK signal in preventing the FLHS progression. We firstly isolated the primary chicken hepatocytes and constructed the FLHS *in vitro* model induced by palmitic acid plus oleic acid (PO), and found that the phosphorylated AMPK (Thr172) protein level was markedly reduced in PO-stimulated primary chicken hepatocytes. More importantly, we found that blocked AMPK signal by AMPK inhibitor compound C exacerbated the lipid metabolism disorders, oxidative stress and inflammatory response triggered by PO in primary chicken hepatocytes; conversely, the lipid metabolism disorders, oxidative stress and inflammatory response challenged by PO were obviously alleviated by activation of AMPK signal using AMPK activator AICAR in hepatocytes. Mechanistically, the mitigative effects of AMPK signal on lipid metabolism disorders, oxidative stress and inflammatory response triggered by PO are achieved through activating the nuclear factor erythroid 2-related factor 2 (NRF-2)/kelch-like ECH-associated protein 1 (KEAP1) pathway and inhibiting the NF-κB pathway in primary chicken hepatocytes. Based on the above results, we speculated that AMPK may be a potential target factor for the prevention of FLHS in laying hens; and these results in present study not only provide a theoretical basis for understand the pathogenesis of FLHS, but also provide a potential guide direction for the prevention of FLHS.

## Materials and methods

### Reagents

The AMPK activator 5-aminoimidazole-4-carboxamide1-β-d-ribofuranoside (AICAR) and AMPK inhibitor 6-(4-[2-piperidin-1-ylethoxy]phenyl)-3-pyridin-4-ylpyrazolo [1,5-a]pyrimidine (compound C, CC) were purchased from Selleck Chemicals (Houston, TX, United States). The commercial assay kits of malondialdehyde (MDA) content, total antioxidant capacity (T-AOC), superoxide dismutase (SOD) activity and catalase (CAT) activity were purchased from Nanjing Jiancheng Biotechnology Institution (Nanjing, China). The peroxidase (POD) activity assay kit was purchased from Beijing Solarbio Science and Technology Co., Ltd. (Beijing, China).

### Isolation, culture and treatment of primary chicken hepatocytes

The isolation and culture of primary chicken hepatocytes were mainly referred to the previous description (Kennedy et al., 1993). Briefly, liver tissues of 9-day-old chicken embryo were collected under sterile conditions, and then cut into piece and digested with 0.25% trypsin for 10–15 min. The suspension containing hepatocytes was passed through a 150 μm-mesh and centrifuged at 600 rpm for 5 min. Subsequently, the primary chicken hepatocytes were seeded in 6-well (2×10^6^ cells/well) or 96-well (1×10^5^ cells/well) culture plates with serum-free M199 medium (Hyclone Laboratories Inc., United States).

The primary chicken hepatocytes were pretreated with dimethyl sulfoxide (DMSO), 10 μM compound C or 1 mM AICAR for 1 h, and then stimulated with 0.75% bovine serum albumin (BSA) or 0.75 mM PO mixture (palmitic acid: oleic acid = 0.25 mM: 0.5 mM) for another 24 h. The compound C and AICAR were prepared in free serum M199 mediums containing less than 0.1% DMSO. PO mixture was prepared in free serum M199 mediums containing 0.75% BSA (Gómez-Lechón et al., 2007).

### Intracellular lipid analysis

The contents of triglyceride (TG) and total cholesterol (TC) in primary chicken hepatocytes were determined using the commercial assay kits (Nanjing Jiancheng Biotechnology Institution, China) according to the introduction of manufacturer.

### Oxidative damage and antioxidant analysis

After treatment, the hepatocytes were collected and broken by ultrasound at 4°C; then, the supernatant was collected for further analysis of MDA content, T-AOC, and the activities of SOD, POD or CAT using the commercial assay kits according to the introduction of manufacturer. Briefly, for the measurement of MDA content, the samples, blank or standard were mixed with MDA working solution and incubated at 96°C for 40°min, and then cooled by flowing water; after centrifuged at 4,000 rpm for 10°min, and the absorbance value at 530 nm was detected using the microplate reader (Bio-Rad, United States). For the measurement of T-AOC, the samples, blank or standard were mixed with T-AOC detection working solution and incubated for 6 min at room temperature; then, the absorbance value at 405 nm was detected using the microplate reader. For the measurement of SOD activity, the samples, samples blank, contrast or contrast blank were mixed with SOD detection working solution and incubated for 20 min at 37°C; then, the absorbance value at 450 nm was detected using the microplate reader. For the measurement of POD activity, the samples were mixed with POD detection working solution and incubated for 30 and 90 s at room temperature; then, the absorbance value at 405 nm was detected using the microplate reader. For the measurement of CAT activity, the samples or contrast were mixed with CAT detection working solution and incubated for 1 min at 37°C; then, the absorbance value at 405 nm was detected using the microplate reader. The MDA contents, T-AOC, and the activity of SOD, POD or CAT in primary chicken hepatocytes were calculated and calibrated by protein concentration.

### Nile Red staining

Primary chicken hepatocytes were stained with Nile Red (0.05 mg/ml; Sigma-Aldrich, United States) for 20 min and washed twice with PBS. The lipid droplets showed red fluorescence and were photographed using a fluorescence microscope (Invitrogen, CA, United States). Then, we randomly selected images of four biological replicates (similar number of cells in the field of view) and quantified the images using ImageJ and SPSS 20.0 software.

### Reactive oxygen species and mitochondrial reactive oxygen species analysis

The levels of reactive oxygen species (ROS) and mitochondrial reactive oxygen species (mtROS) in chicken hepatocytes were determined according to our recent description (Li et al., 2021). Briefly, the hepatocytes were incubated with a 10 μM DCFH-DA probe (Beyotime Institute of Biotechnology, China) or 2.5 μM MitoSOX Red probe (Thermo Fisher Scientific, United States) at 37°C for 25 min, and then washed twice with PBS to remove the unbound fluorescent probe. The intracellular ROS showed green fluorescence and the mtROS presented red fluorescence, and ROS or mtROS were photographed using a fluorescence microscope (Invitrogen, CA, United States). Then, we randomly selected images of four biological replicates (similar number of cells in the field of view) and quantified the images using Image J and SPSS 20.0 software.

### Mitochondrial membrane potential analysis

The primary chicken hepatocytes were seed in 96-well culture plates and incubated with JC-1 dyeing liquid (Beyotime Institute of Biotechnology, Shanghai, China) for 20 min. Then, the cells were washed twice with PBS to remove the unbound fluorescent probe and photographed using a fluorescence microscope (Invitrogen, CA, United States). When the mitochondrial membrane potential (MMP) is high, the JC-1 accumulates in the matrix of mitochondria to form polymers (J-aggregates), which can produce red fluorescence. As the MMP is low, the JC-1 cannot aggregate in the mitochondrial matrix; at this time, the JC-1 is a monomer (JC-1 monomers) and can emit bright green fluorescence. After photographed using a fluorescence microscope, we randomly selected images of four biological replicates (similar number of cells in the field of view) and quantified the images using ImageJ and SPSS 20.0 software.

### Real-time quantitative PCR (RT-qPCR)

Total RNA was isolated from primary chicken hepatocytes using the Trizol reagent (Sigma-Aldrich, United States), and then reverse transcribed into cDNA using the HiScript® reverse transcription kit (Vazyme Biotech Co., Ltd., China). The cDNA samples were detected by using SYBR Green PCR Master Mix Kit (Roche, Switzerland). RT-qPCR was performed using the ABI 7500 Real-time Detection System (Applied Biosystems, United States). The mRNA levels were calculated and normalized to β-actin with the 2^−ΔΔCT^ method. The primers of fatty acid translocase (*CD36*), sterol regulatory element binding protein 1c (*SREBP-1c*), acetyl-CoA carboxylase α (*ACCα*), fatty acid synthase (*FASN*), peroxisome proliferator activated receptor α (*PPARα*), carnitine palmitoyl transterase-1 (*CPT-1*), interleukin 6 (*IL-6*), tumor necrosis factor α (*TNF-*α) and *β-actin* were synthesized by GenScript Biotechnology Co., Ltd. (Nanjing, China); all primer sequences of RT-qPCR are showed in Table 1.

**TABLE 1:** Primer sequences of targeted genes and β-actin.

Gene	GenBank accession number	Primer sequences (5′–3′)	Orientation	Product size (bp)
*β-actin*	L08165	TGCGTGACATCAAGGAGAAG TGCCAGGGTACATTGTGGTA	Forward Reverse	300
*CD36*	NM_001030731	GGGCATCATTTCCTCCATTT GGGCTCAGACCTTCAACATC	Forward Reverse	334
*SREBP-1c*	AY029224	GTCGGCGATCCTGAGGAA CTCTTCTGCACGGCCATCTT	Forward Reverse	105
*ACCα*	J03541	GTTGTGGTTGGCAGAGCAAG GCACCAAACTTGAGCACCTG	Forward Reverse	284
*FASN*	NM_205155	TGAAGGACCTTATCGCATTGC GCATGGGAAGCATTTTGTTGT	Forward Reverse	96
*PPARα*	AF470455	CAAACCAACCATCCTGACGAT GGAGGTCAGCCATTTTTTGGA	Forward Reverse	64
*CPT-1*	AY675193	GGGTTGCCCTTATCGTCACA TACAACATGGGCTTCCGTCC	Forward Reverse	151
*IL-6*	*NM_204628.1*	*GAGGAGAAATGCCTGACGA* *AGGATTGTGCCCGAACTAA*	*Forward* *Reverse*	366
*TNF-*α	NM_204267.1	GCATTTGGAAGCAGCGTTTG GGTTGTGGGACAGGGTAGGG	Forward Reverse	211

### Western blot

The methods of protein extraction and western blot analysis of chicken hepatocytes was referred to our recent description (Li et al., 2021). Briefly, the primary chicken hepatocytes were homogenized in the RIPA lysis buffer (Beyotime Institute of Biotechnology, Shanghai, China) with 1% protease and phosphatase inhibitors (Beyotime Institute of Biotechnology, Shanghai, China). The protein concentration of lysate was determined using a BCA protein assay kit (Beyotime Institute of Biotechnology, Shanghai, China) according to the introduction of manufacturer. 15 μg extracted protein samples were separated by the 10% sodium dodecyl sulfate-polyacrylamide gel electrophoresis (SDS-PAGE) gel and then transferred onto polyvinylidene fluoride (PVDF) membrane (Millipore, Bedford, MA, United States). After blocking with 5% skimmed milk at room temperature for 2 h, the membranes were incubated with rabbit antibodies against the nuclear factor erythroid 2-related factor 2 (NRF-2), NAD (P) H: quinone oxidoreductase 1 (NQO1), fatty acid synthase (FASN) (Bioworld Technology, St. Louis Park, MN, United States), heme oxygenase 1 (HO-1), IκBα, PCNA, (Proteintech Group, Wuhan, China), phospho (*p*)-IκBα (Affinity Biosciences, China), p-NF-κBα p65, NF-κBα p65, acetyl-CoA carboxylase (ACCα), p-ACCα (Ser79), AMPKα, p-AMPKα (Thr172) (Cell Signaling Technology, United States) and β-actin (ABclonal Technology, Wuhan, China) overnight at 4°C. Then, membranes were washed with TBST for 1 h at room temperature, and incubated with HRP goat anti-rabbit IgG (Proteintech Group, Wuhan, China) for another 2 h. Proteins were visualized using the clarity ECL chemiluminescent substrate (Tanon Technology Co., Ltd., Shanghai, China), and the quantification was performed by ImageJ software. The PCNA or β-actin were used to normalize the nuclear protein or total protein.

### Statistical analysis

All data are expressed as the mean value with the standard errors of the mean (mean ± SEM), and all statistical analyses are computed by using the SPSS 20.0 software (IBM Corporation, United States) and GraphPad Prism 8 (GraphPad, United States). Differences between two treatment groups were evaluated by two-tailed Student’s t-test, and comparisons among multiple groups (no less than three groups) were analyzed by one-way analysis of variance (ANOVA) followed by post-hoc tests. Significance was considered at *p* < 0.05.

## Results

### Palmitic acid plus oleic acid-challenged causes the inhibition of hepatic AMP-activated protein kinase signal in primary chicken hepatocytes

**FIGURE 1:**
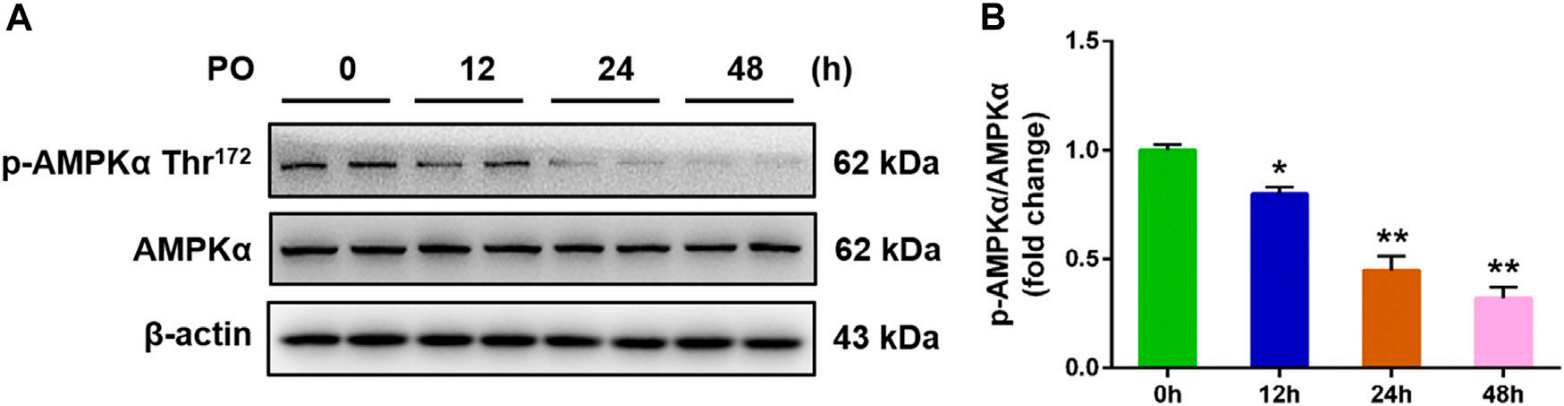
PO stimulation causes the down-regulation on the phosphorylated AMPK protein level in primary chicken hepatocytes. The primary chicken hepatocytes were stimulated with 0.75 mmol PO for 0–48 h. **(A)** Immunoblot of phosphorylated AMP-activated protein kinase α (AMPKα) (Thr172) and AMPKα in primary chicken hepatocytes; **(B)** Statistical analysis of phospho (*p*)-AMPKα (Thr172) and AMPKα protein level. Values represent the means ± SE. *p < 0.05 and **p < 0.01, compared with the control group.

To investigate the relevance between AMPK signal and metabolic disorders, the phosphorylated AMPKα (Thr172) protein level was detected in PO-induced primary chicken hepatocytes. The results illustrated that the phosphorylation protein level of AMPKα (Thr172) was down-regulated in PO-stimulated primary chicken hepatocytes with a time-dependent manner (Figures 1A,B), which suggested that the inhibition of AMPK signal may be associated with the development of obesity-induced pathologies and that p-AMPKα (Thr172) may act a crucial role in this process.

### Inhibited AMP-activated protein kinase signal exacerbates lipid accumulation challenged by palmitic acid plus oleic acid in primary chicken hepatocytes

**FIGURE 2:**
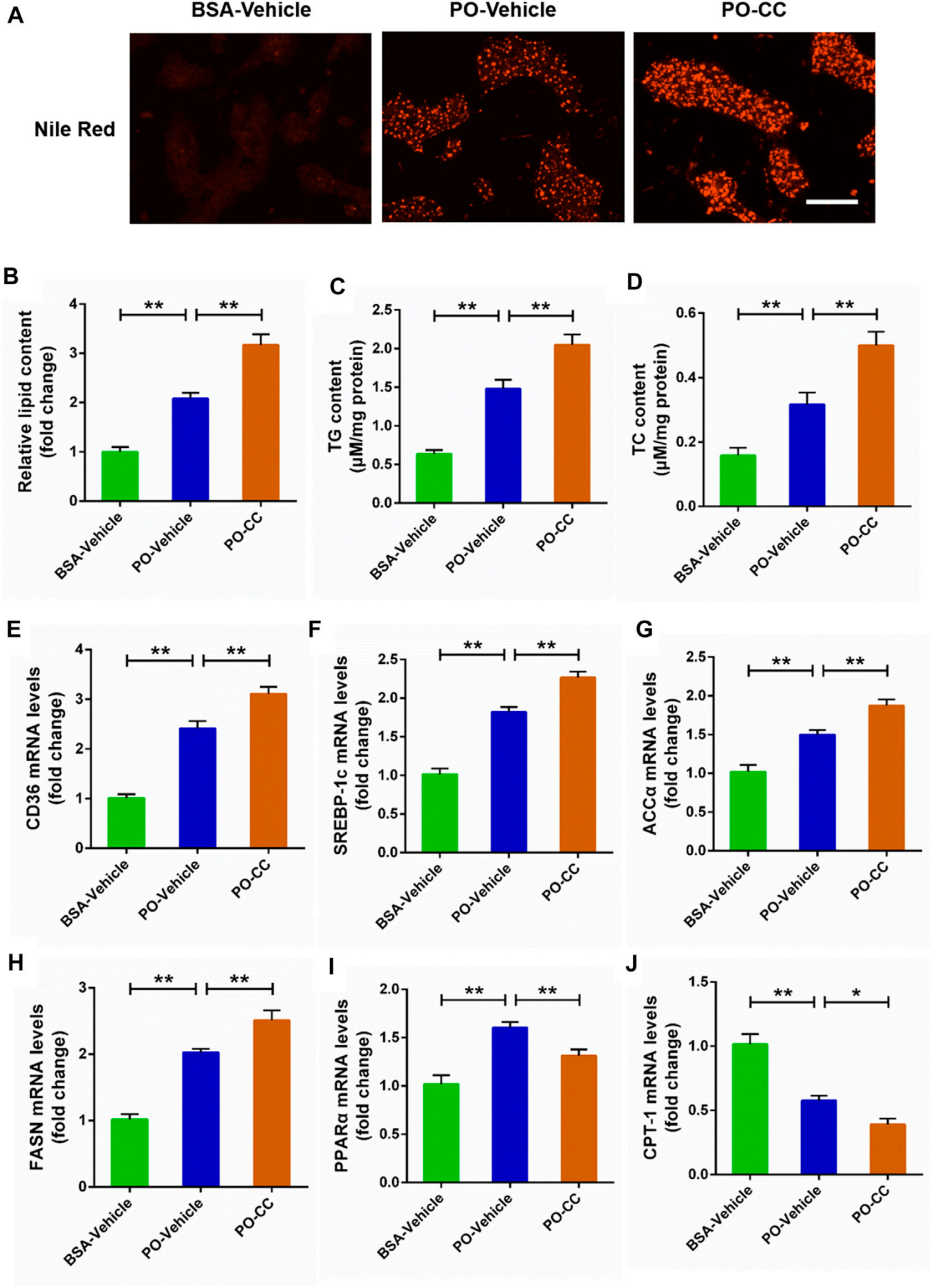
Compound C exacerbates the lipid metabolism disorders induced by PO in primary chicken hepatocytes. The primary chicken hepatocytes were pre-treated with/without 10 μM AMPK inhibitor compound C for 1 h, and then stimulated with/without 0.75 mmol PO for another 24 h. **(A)** Photography of lipid droplet accumulation by Nile Red staining (scale bars, 100 μm); **(B)** Relative of lipid droplet content; **(C)** Triglyceride (TG) content; **(D)** Total cholesterol (TC) content; **(E)** Fatty acid translocase (CD36) mRNA levels; **(F)** Sterol regulatory element binding protein 1c (SREBP-1c) mRNA levels; **(G)** Acetyl-CoA carboxylase α (ACCα) mRNA levels; **(H)** Fatty acid synthase (FASN) mRNA levels; **(I)** Peroxisome proliferator activated receptor α (PPARα) mRNA levels; **(J)** Carnitine palmitoyl transterase-1 (CPT-1) mRNA levels. Values represent the means ± SE. *p < 0.05 and **p < 0.01, significantly different between the indicated treatment groups.

To determine the role of AMPK signal on lipid metabolism disorders triggered by PO, the primary chicken hepatocytes were pre-treated with AMPK inhibitor compound C. Nile Red staining analysis showed that the excessive accumulation of lipid droplets induced by PO was markedly exacerbated in hepatocytes pre-treated with compound C (*p* < 0.01) (Figures 2A,B). Meanwhile, compound C pre-treatment aggravated cellular lipid level that presented as the increases of TG and TC contents in hepatocytes (*p* < 0.01) (Figures 2C,D). Then, we further analyzed the effect of compound C on lipid metabolism related factors expression in PO-stimulated primary chicken hepatocytes, and found that compound C pre-treatment strengthened the increases of the lipid transport (*CD36*) and fatty acids synthesis (*ACCα*, *FASN*, and *SREBP-1c*) related gene expression induced by PO in hepatocytes (*p* < 0.01) (Figures 2E–H). However, compound C markedly inhibited lipid oxidation (*PPARα* and *CPT-1*) related gene expression level in PO-stimulated primary chicken hepatocytes (*p* < 0.01) (Figures 2I,J). These data suggested that the inhibition of AMPK signal exacerbates the excessive lipid accumulation challenged by PO in primary chicken hepatocytes.

### Activated AMP-activated protein kinase signal protects against excessive lipid accumulation challenged by palmitic acid plus oleic acid in primary chicken hepatocytes

**FIGURE 3:**
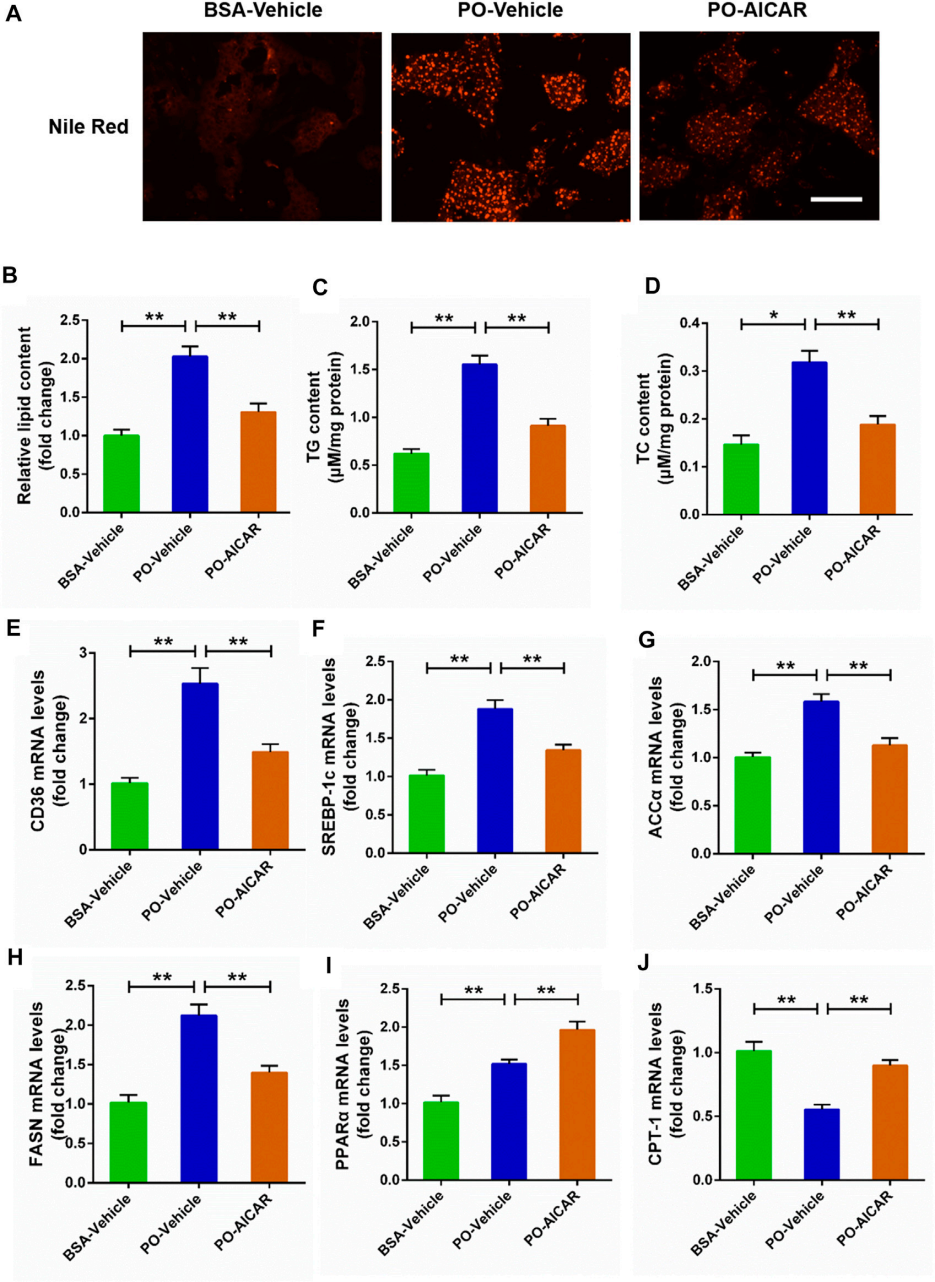
AICAR alleviates the lipid metabolism disorders induced by PO in primary chicken hepatocytes. The primary chicken hepatocytes were pre-treated with/without 1 mM AMPK activator AICAR for 1 h, and then stimualted with/without 0.75 mmol PO for another 24 h. **(A)** Photography of lipid droplet accumulation by Nile Red staining (scale bars, 100 μm); **(B)** Relative of lipid droplet content; **(C)** Triglyceride (TG) content; **(D)** Total cholesterol (TC) content; **(E)** Fatty acid translocase (CD36) mRNA levels; **(F)** Sterol regulatory element binding protein 1c (SREBP-1c) mRNA levels; **(G)** Acetyl-CoA carboxylase α (ACCα) mRNA levels; **(H)** Fatty acid synthase (FASN) mRNA levels; **(I)** Peroxisome proliferator activated receptor α (PPARα) mRNA levels; **(J)** Carnitine palmitoyl transterase-1 (CPT-1) mRNA levels. Values represent the means ± SE. *p < 0.05 and **p < 0.01, significantly different between the indicated treatment groups.

For further certified the beneficial role of AMPK signal on the lipid metabolism disorders induced by PO, the primary chicken hepatocytes were pre-treated with AMPK activator AICAR. The results showed that AICAR pretreatment significantly alleviated the excessive accumulation of lipid droplets triggered by PO in hepatocytes (*p* < 0.01) (Figures 3A,B). Meanwhile, AICAR pretreatment remarkably attenuated the increases of TG and TC contents induced by PO in hepatocytes (Figures 3C,D). In addition, AICAR pretreatment evidently decreased PO-induced upregulation of genes expression level that participates in lipid transport (*CD36*) and fatty acids synthesis (*ACCα*, *FASN*, and *SREBP-1c*) (*p* < 0.01) (Figures 3E–H). In contrast, AICAR pretreatment markedly increased the genes expression level that related to lipid oxidation (*PPARα* and *CPT-1*) in PO-stimulated primary chicken hepatocytes (*p* < 0.01) (Figures 3I,J). These results indicated that activation of AMPK signal alleviates the excessive lipid accumulation triggered by PO in primary chicken hepatocytes.

### AMP-activated protein kinase inhibits the expression of lipid synthesis related factors in palmitic acid plus oleic acid-stimulated primary chicken hepatocytes

**FIGURE 4:**
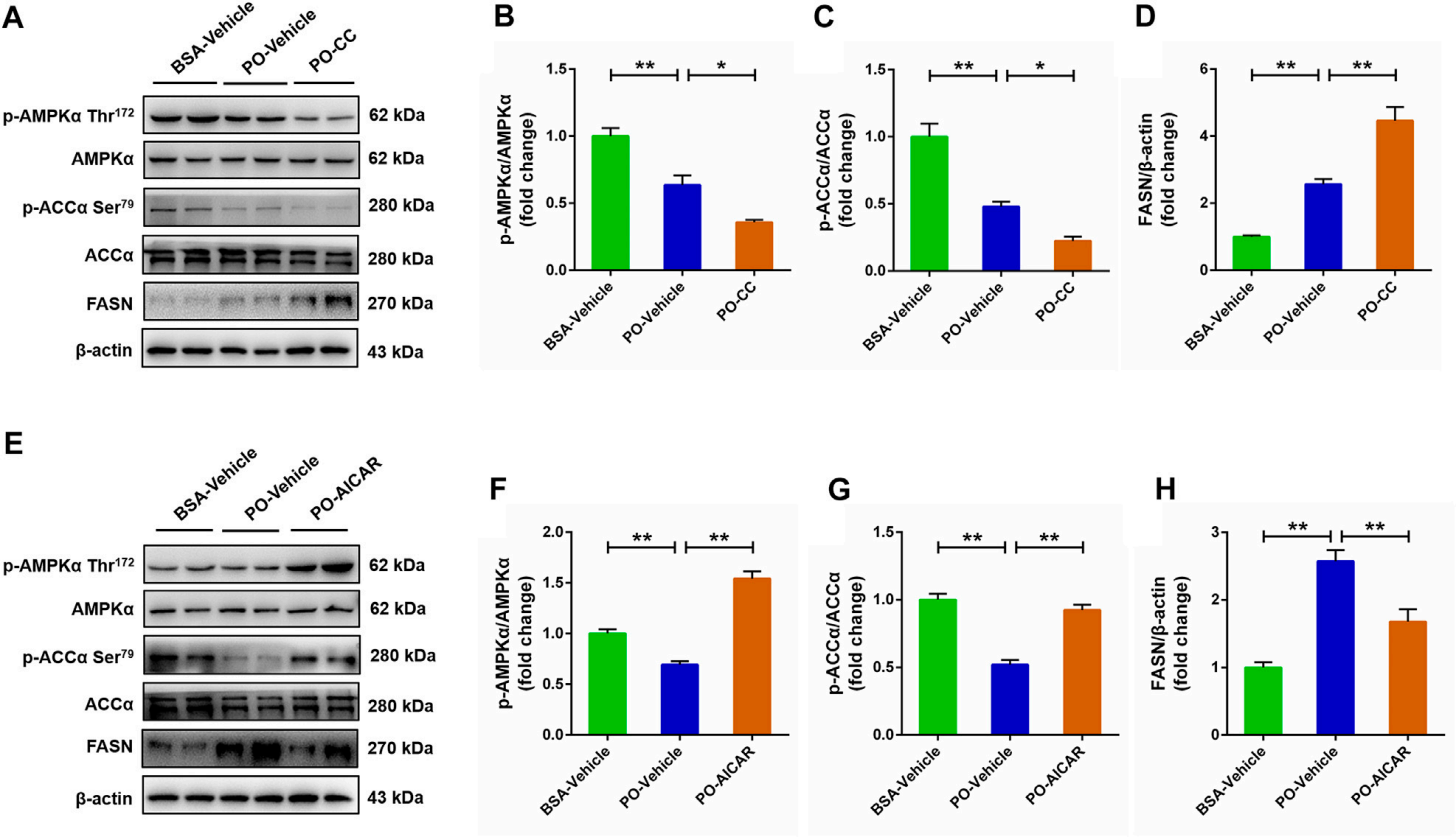
AMPK regulates the lipid synthesis related factors expression level in PO-stimulated primary chicken hepatocytes. The primary chicken hepatocytes were pre-treated with/without 10 μM AMPK inhibitor compound C for 1 h, and then stimulated with/without 0.75 mmol PO for another 24 h. **(A)** Immunoblot of phosphorylated AMP-activated protein kinase α (AMPKα) (Thr172), AMPKα, phospho (*p*)-acetyl-CoA carboxylase α (p-ACCα), ACCα and fatty acid synthase (FASN). **(B)** Statistical analysis of p-AMPKα (Thr172)/AMPKα; **(C)** Statistical analysis of p-ACCα/ACCα; **(D)** Statistical analysis of FASN/β-actin. The primary chicken hepatocytes were pre-treated with/without 1 mM AMPK activator AICAR for 1 h, and then stimulated with/without 0.75 mmol PO for another 24 h. **(E)** Immunoblot of p-AMPKα (Thr172), AMPKα, p-ACCα, ACCα and FASN. **(F)** Statistical analysis of p-AMPKα/AMPKα; **(G)** Statistical analysis of p-ACCα/ACCα; **(H)** Statistical analysis of FASN/β-actin. Values represent the means ± SE. *p < 0.05 and **p < 0.01, significantly different between the indicated treatment groups.

For further certified the role of AMPK signal in regulating lipid metabolism in PO-stimulated primary chicken hepatocytes, the protein expression level of lipid synthesis related factors was analyzed using western blot. As shown in Figures 4A–D, phosphorylation of AMPKα (Thr172) and ACCα protein level were decreased, but the FASN protein level was increased in PO-stimulated primary chicken hepatocytes, and these effects were exacerbated as the hepatocytes pre-treated with compound C (*p* < 0.01) (Figures 4A–D). In contrast, PO-induced the downregulation of phosphorylation of AMPKα (Thr172) and ACCα protein level, and the upregulation of FASN protein level were significantly alleviated in primary chicken hepatocytes by AICAR (*p* < 0.01) (Figures 4E–H). These data suggested that activated AMPK signal inhibits the lipid synthesis related factors protein level in PO-stimulated primary chicken hepatocytes.

### Inhibited AMP-activated protein kinase signal exacerbates mitochondrial oxidative stress triggered by palmitic acid plus oleic acid in primary chicken hepatocytes

**FIGURE 5:**
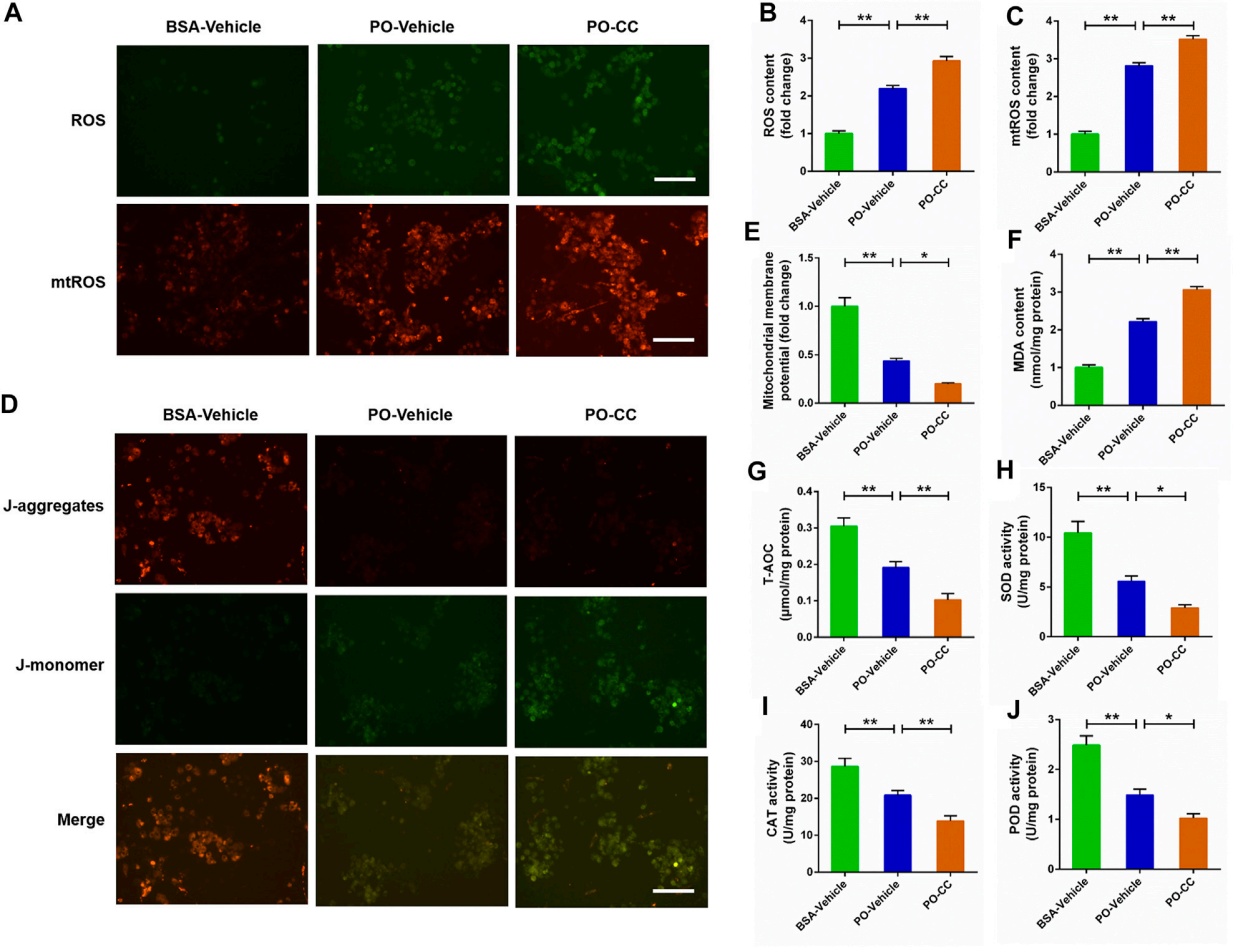
Inhibition of AMPK signal exacerbates the oxidative stress challenged by PO in primary chicken hepatocytes. The primary chicken hepatocytes were pre-treated with/without 10 μM AMPK activator compound C for 1 h, and then stimulated with/without 0.75 mmol PO for another 24 h. **(A)** Fluorescence photography of reactive oxygen species (ROS) (scale bars, 100 μm) and mitochondrial ROS (mtROS) (scale bars, 100 μm); **(B)** Statistical analysis of ROS level; **(C)** Statistical analysis of mtROS level; **(D)** Fluorescence photography of mitochondrial membrane potential (MMP) were detected by JC-1 (scale bars, 100 μm); **(E)** Statistical analysis of MMP; **(F)** Malondialdehyde (MDA) content; **(G)** total antioxidant capacity (T-AOC); **(H)** Superoxide dismutase (SOD) activity; **(I)** Catalase (CAT) activity; **(J)** Peroxidase (POD) activity. Values represent the means ± SE. *p < 0.05 and **p < 0.01, significantly different between the indicated treatment groups.

To determine the role of AMPK pathway on mitochondrial oxidative stress induced by PO, the primary chicken hepatocytes were pre-treated with AMPK inhibitor compound C. The results showed that PO-induced the increases of ROS and mtROS contents, and the decrease of MMP level were markedly exacerbated in primary chicken hepatocytes pre-treated with compound C (*p* < 0.01) (Figures 5A–E). Meanwhile, compound C pretreatment remarkably aggravated the increase of MDA content challenged by PO in hepatocytes (*p* < 0.01) (Figure 5F). In contrast, PO-induced decreases in the T-AOC, SOD, POD and CAT activities were also exacerbated in hepatocytes by compound C pretreatment (*p* < 0.05) (Figures 5G–J). These results implied that blocking the activation of AMPK signal by compound C exacerbates mitochondrial oxidative stress triggered by PO in primary chicken hepatocytes.

### Activated AMP-activated protein kinase signal alleviates mitochondrial oxidative stress triggered by palmitic acid plus oleic acid in primary chicken hepatocytes

**FIGURE 6:**
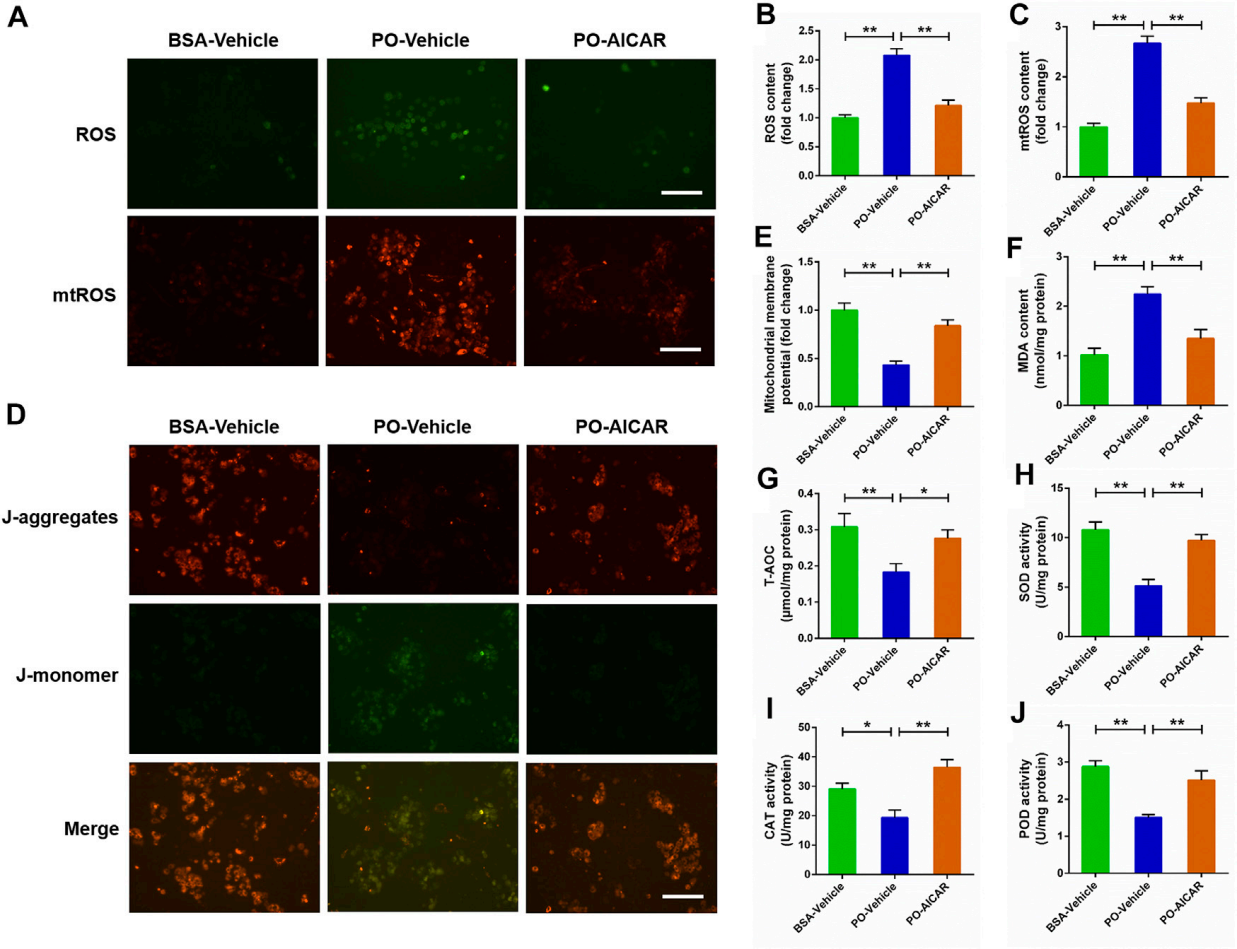
Activation of AMPK signal alleviates the oxidative stress challenged by PO in primary chicken hepatocytes. The primary chicken hepatocytes were pre-treated with/without 1 mM AMPK activator AICAR for 1 h, and then stimulated with/without 0.75 mmol PO for another 24 h. **(A)** Fluorescence photography of reactive oxygen species (ROS) (scale bars, 100 μm) and mitochondrial ROS (mtROS) (scale bars, 100 μm); **(B)** Statistical analysis of ROS level; **(C)** Statistical analysis of mtROS level; **(D)** Fluorescence photography of mitochondrial membrane potential (MMP) were detected by JC-1 (scale bars, 100 μm); **(E)** Statistical analysis of MMP; **(F)** Malondialdehyde (MDA) content; **(G)** total antioxidant capacity (T-AOC); **(H)** Superoxide dismutase (SOD) activity; **(I)** Catalase (CAT) activity; **(J)** Peroxidase (POD) activity. Values represent the means ± SE. *p < 0.05 and **p < 0.01, significantly different between the indicated treatment groups.

For further certified the beneficial effect of AMPK signal on PO-induced mitochondrial oxidative stress, the primary chicken hepatocytes were pre-treated with AMPK activator AICAR. As shown in Figures 6A–E, AICAR pretreatment significantly alleviated PO-induced increases of ROS and mtROS contents, and the decrease of MMP level in hepatocytes (*p* < 0.01). Moreover, the increase of MDA content induced by PO was markedly ameliorated in hepatocytes by AICAR pretreatment (*p* < 0.01) (Figure 6F). Conversely, AICAR pretreatment significantly enhanced the T-AOC, SOD, POD and CAT activities in PO-stimulated hepatocytes (*p* < 0.05) (Figures 6G–J). These data revealed that activation of AMPK signal by AICAR alleviates the mitochondrial oxidative stress triggered by PO in primary chicken hepatocytes.

### Activated AMP-activated protein kinase induces the activation of nuclear factor erythroid 2-related factor 2/kelch-like ECH-associated protein 1 signaling pathway in palmitic acid plus oleic acid-stimulated primary chicken hepatocytes

**FIGURE 7:**
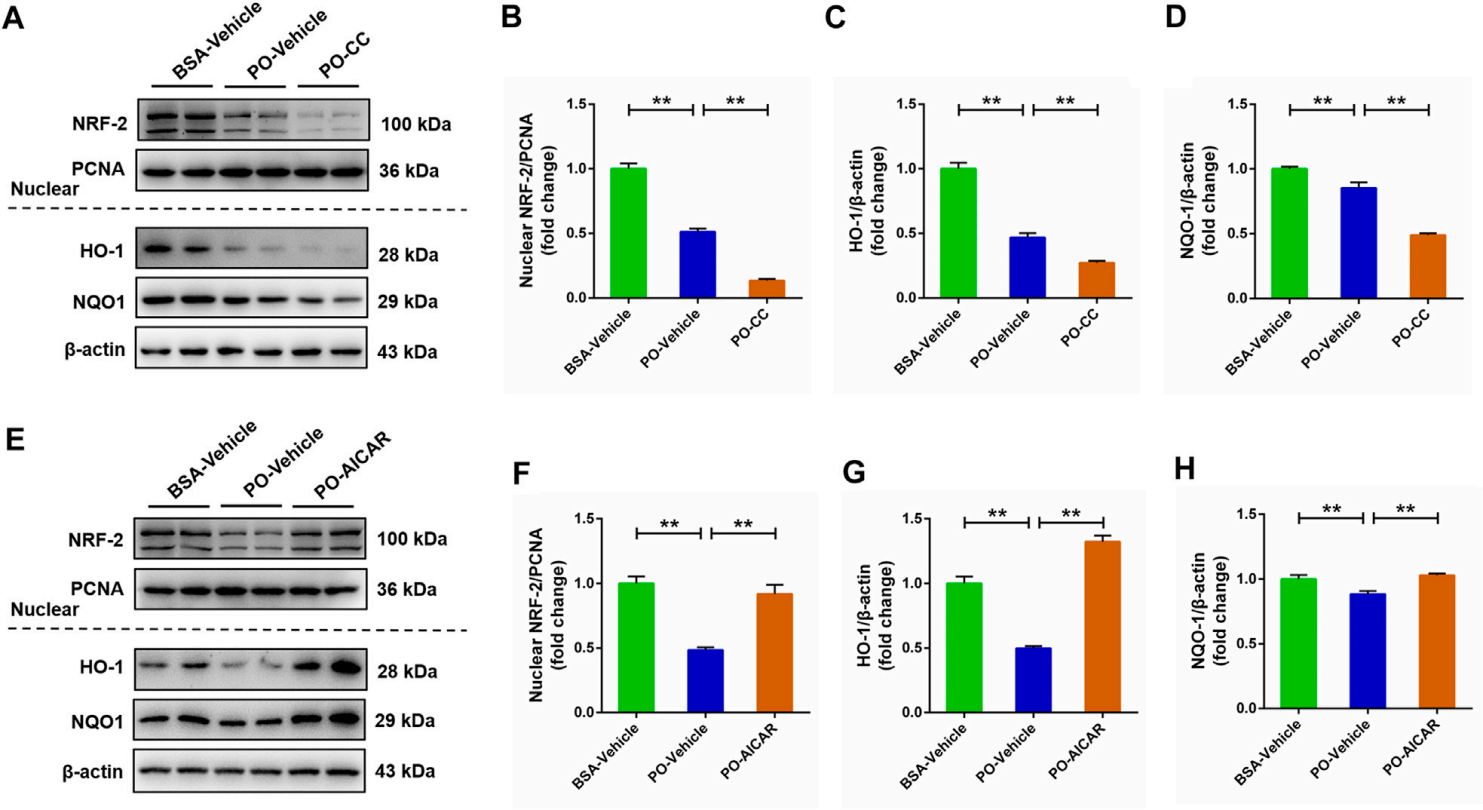
AMPK regulates the NRF-2 signaling pathways in PO-induced oxidative stress of hepatocytes. The primary chicken hepatocytes were pre-treated with/without 10 μM AMPK inhibitor compound C for 1 h, and then stimulated with/without 0.75 mmol PO for another 24 h. **(A)** Immunoblot of nuclear factor erythroid 2-related factor 2 (NRF-2), heme oxygenase 1 (HO-1) and NAD (P) **(H)** quinone oxidoreductase 1 (NQO1); **(B)** Statistical analysis of nuclear NRF-2 protein level; **(C)** Statistical analysis of HO-1 protein level; **(D)** Statistical analysis of NQO1 protein level. The primary chicken hepatocytes were pre-treated with/without 1 mM AMPK activator AICAR for 1 h, and then stimulated with/without 0.75 mmol PO for another 24 h. **(E)** Immunoblot of nuclear NRF-2, HO-1 and NQO1; **(F)** Statistical analysis of nuclear NRF-2 protein level; **(G)** Statistical analysis of HO-1 protein level; **(H)** Statistical analysis of NQO1 protein level. Values represent the means ± SE. *p < 0.05 and **p < 0.01, significantly different between the indicated treatment groups.

The NRF-2/KEAP1 signaling pathway has been indicated to be associated with oxidative stress. Thus, we subsequently evaluated whether NRF-2/KEAP1 signaling pathway was regulated by AMPK in PO-stimulated primary chicken hepatocytes. Compared with BSA-vehicle group, PO stimulation significantly decreased the nuclear NRF-2, HO-1 and NQO-1 protein levels in hepatocytes, and these inhibitory effects were obviously exacerbated in PO-stimulated hepatocytes pre-treated with compound C (*p* < 0.01) (Figures 7A–D). However, PO-induced downregulation of nuclear NRF-2, HO-1 and NQO-1 protein levels were significantly alleviated in hepatocytes by AICAR pretreatment (*p* < 0.01) (Figures 7E–H). These findings suggested that the NRF-2/KEAP1 signaling pathway is regulated by AMPK in PO-induced oxidative stress of hepatocytes.

### Activated AMP-activated protein kinase suppresses NF-κB signaling pathway to relieve inflammation response induced by palmitic acid plus oleic acid in primary chicken hepatocytes

**FIGURE 8:**
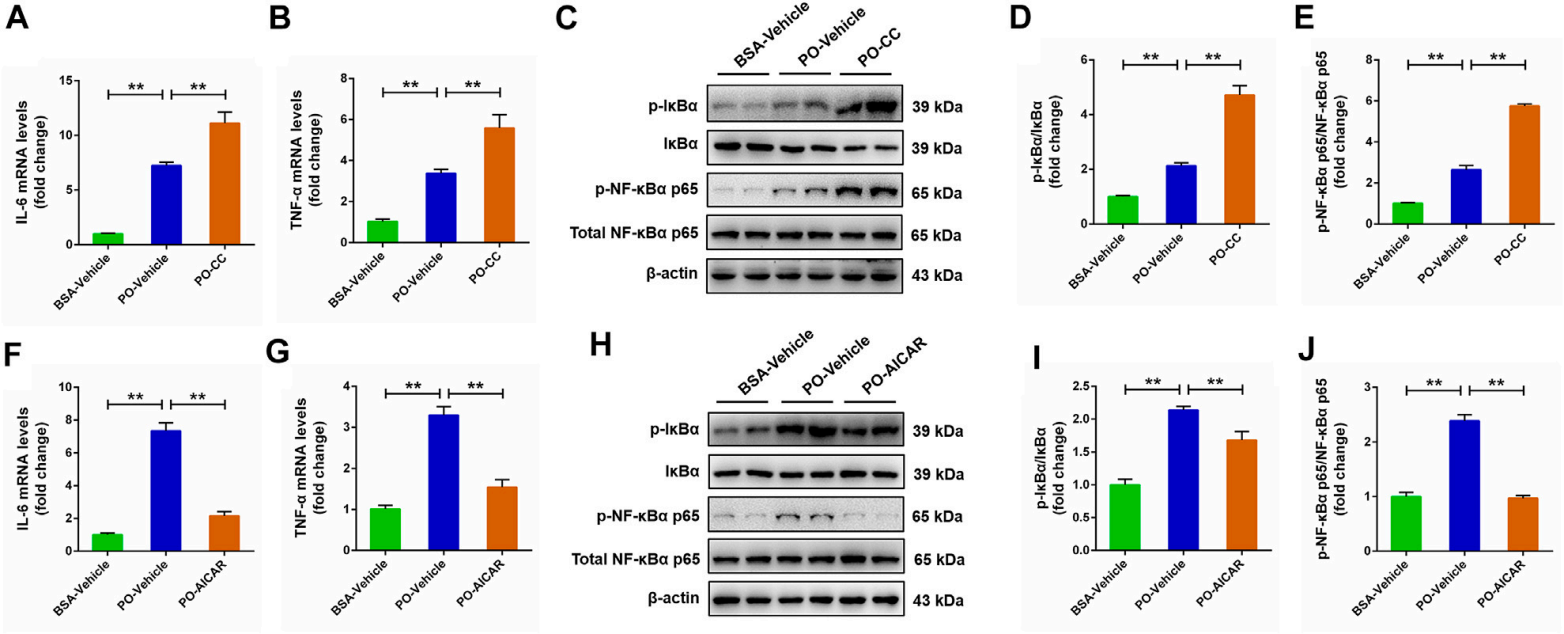
AMPK regulates pro-inflammatory mediator expression level and NF-κB signaling pathway in PO-stimulated primary chicken hepatocytes. The primary chicken hepatocytes were pre-treated with/without 10 μM AMPK inhibitor compound C for 1 h, and then stimulated with/without 0.75 mmol PO for another 24 h. **(A)** Interleukin 6 (IL-6) mRNA levels; **(B)** Tumor necrosis factor α (TNF-α) mRNA levels; **(C)** Immunoblot of phospho (*p*)-IκBα, IκBα, p-NF-κBα p65 and NF-κBα p65; **(D)** Statistical analysis of p-IκBα/IκBα; **(E)** Statistical analysis of p-NF-κBα p65/NF-κBα p65. The primary chicken hepatocytes were pre-treated with/without 1 mM AMPK activator AICAR for 1 h, and then stimulated with/without 0.75 mmol PO for another 24 h. **(F)** IL-6 mRNA levels; **(G)** TNF-α mRNA levels; **(H)** Immunoblot of p-IκBα, IκBα, p-NF-κBα p65 and NF-κBα p65; **(I)** Statistical analysis of p-IκBα/IκBα; **(J)** Statistical analysis of p-NF-κBα p65/NF-κBα p65. *p < 0.05 and **p < 0.01, significantly different between the indicated treatment groups.

Activated AMPK contributes to the regulation of NF-κB signaling pathway, which is closely related to the pathogenesis of a series of metabolic disorders. This relationship prompted us to investigate whether the NF-κB signaling pathway is involved in AMPK-mediated metabolic disorders. The results showed that PO stimulation caused a significantly rise on the pro-inflammatory mediators (*IL-6* and *TNF-*α) level in primary chicken hepatocytes, and the increases of *IL-6* and *TNF-*α challenged by PO was markedly exacerbated in hepatocytes by compound C pretreatment (*p* < 0.01) (Figures 8A,B). Meanwhile, the increases of phosphorylation of NF-κBα p65 and IκBα protein level induced by PO were also exacerbated in hepatocytes pre-treated with compound C (*p* < 0.01) (Figures 8C–E). Conversely, AICAR pretreatment significantly alleviated PO-induced upregulation of the proinflammatory mediators (*IL-6* and *TNF-*α) level in primary chicken hepatocytes (*p* < 0.01) (Figures 8F,G); similarly, PO-induced upregulation in the phosphorylation of NF-κBα p65 and IκBα protein level were also significantly alleviated in hepatocytes by AICAR pretreatment (*p* < 0.01) (Figures 8H–J). These data suggested that the NF-κB signaling pathway is mediated by AMPK in PO-induced inflammation response of hepatocytes.

## Discussion

FLHS is a common hepatic metabolic disease that mainly caused by the imbalance between energy expenditure and consumption. Interestingly, both FLHS in laying hens and NAFLD in humans are have the similarity pathology features and clinical symptoms that characterized as the lipid metabolism disorders, inflammatory response and oxidative stress in the liver, and also simultaneously accompanied by obesity and dyslipidemia. Many researchers, including our laboratory, have found that the activation of AMPK signal can protect against the occurrence and development of NAFLD that presented as mitigative effect on the hepatic steatosis, inflammatory response and oxidative stress in mammals (Hu et al., 2021; Li et al., 2021; Wan et al., 2021). However, whether AMPK acts a crucial role in the occurrence and development of FLHS remains elucidated. In current study, we discovered a potential target factor for preventing the occurrence and development of FLHS in laying hens; and further investigation certified that the inhibition of AMPK signal obviously exacerbated the excessive lipid accumulation, oxidative stress and inflammatory response triggered by PO in primary chicken hepatocytes, but activated AMPK signal presented obviously beneficial effects on PO-induced the excessive lipid accumulation, oxidative stress and inflammatory response in hepatocytes. Mechanistically, this study confirmed that AMPK exerts its beneficial role in relieving of lipid metabolism disorders, oxidative stress and inflammatory response triggered by PO by activating the NRF-2/KEAP1 pathway and inhibiting the NF-κB pathway. These data demonstrated that AMPK may be an important potential target factor for the prevention of FLHS occurrence in laying hens.

Our recently study reported that the phosphorylated AMP-activated protein kinase (AMPK) protein level was obviously decreased in the liver of mouse with NAFLD than that of normal liver tissue (Li et al., 2021). AMPK is a major sensor of cellular energy status and that plays a crucial role in regulating lipid metabolism, glucose metabolism, oxidative stress and inflammatory responses in body (Green et al., 2022). It had been indicated that AMPK is a potential target in the treatment and prevention of NAFLD in mammals (Strzyz, 2020). In this study, we found that the phosphorylated AMPK (Thr172) protein level was decreased in PO-stimulated hepatocytes with a time-dependent manner. This result implied that the inhibition of AMPK signal maybe associate with the development of obesity-induced pathologies and which AMPK acts a crucial role in this process.

To investigate the potential target factors in the occurrence and development of FLHS, the *in vitro* model of FLHS was successfully constructed in primary chicken hepatocytes triggered by PO that presented as the excessive lipid accumulation, oxidative stress and inflammatory response. Generally, the cellular lipid content is relatively stable and that is affected by fat synthesis and decomposition. However, as the free fatty acids (FFA), including palmitic acid (PA), oleic acid (OA) and PO (palmitic acid plus oleic acid) entering the cells cannot be decomposed effectively, which can induce the acceleration of fat synthesis, and finally resulting in the excessive accumulation of lipid droplets. In the current study, PO-stimulated markedly caused a rise in the lipid droplet accumulation and the contents of TG and TC in primary chicken hepatocytes; moreover, these effects were obviously exacerbated in PO-stimulated hepatocytes by AMPK inhibitor compound C pre-treatment. In contrast, PO-induced the excessive lipid accumulation was markedly alleviated in hepatocytes by AMPK activator AICAR pretreatment. These results are consist with our previous reported that activation of AMPK signal alleviates FFA-induced the lipid deposition in BRL-3A and L02 hepatocytes (Chu et al., 2022). These data suggested that AMPK signal plays an important role in controlling lipid accumulation in primary chicken hepatocytes.

Many factors related to lipid metabolism, including lipid transport, synthesis and oxidative decomposition of fatty acids, all which play a decisive role in the lipid droplets accumulation. A recent study found that FFA-treatment markedly decreased the phosphorylated AMPK (Thr172) protein level, and which subsequently led to the downregulation of phosphorylated ACC protein level and the upregulation of FASN and SREBP-1 protein level (Kim et al., 2020). In the current study, the phosphorylated AMPK (Thr172) and ACC protein levels were inhibited in PO-stimulated primary hepatocytes by compound C pretreatment; but that were significantly increased in hepatocytes by AICAR pretreatment, which indicated that AMPK signal was inhibited by compound C and activated by AICAR. Furthermore, we found that compound C pretreatment significantly exacerbated PO-induced the upregulation of genes expression level that participating in lipid transport (*CD36*) and fatty acids synthesis (*ACCα*, *FASN* and *SREBP-1c*). In contrast, AICAR pretreatment markedly alleviated PO-induced the upregulation of genes expression level participating in lipid transport (*CD36*) and fatty acids synthesis (*ACCα*, *FASN* and *SREBP-1c*). It has been reported that CD36 is responsible for the transporting of free fatty acids and promoting the *de novo* synthesis of fatty acids under regulation of lipid synthesis related factors, including ACCα, FASN and SREBP-1c expression level in body (Jones and Infante, 2015; Maréchal et al., 2018). Our recent study also found that activation of AMPK signal alleviates the hepatic steatosis by inhibiting the lipid synthesis related factors expression in mice (Li et al., 2021). Thus, these findings implied that AMPK signal exerts a regulative effect on the lipid transport and fatty acids synthesis in PO-stimulated primary chicken hepatocytes. Numerous studies confirmed that activation of AMPK signal can improve the lipid oxidation in mammals (Kim et al., 2020; Li et al., 2021). However, there are few reports about whether AMPK signal can regulate the oxidation of fatty acids in chickens. In this study, compound C pretreatment markedly inhibited the genes expression related to lipid oxidation (*PPARα* and *CPT-1*) in PO-stimulated primary chicken hepatocytes. However, AICAR pretreatment markedly increased the genes expression related to lipid oxidation (*PPARα* and *CPT-1*) in PO-stimulated primary chicken hepatocytes. Taken together, these data indicated that the activation of AMPK signal alleviates excessive lipid accumulation triggered PO through inhibiting of lipid transport and fatty acids synthesis and enhancement of lipid oxidation in primary chicken hepatocytes.

As well known, the lipid metabolism disorders, oxidative stress and inflammatory response in the liver are the typical pathology features and clinical symptoms in FLHS of laying hens. Our recent study demonstrated that AMPK can regulate the oxidative stress in PA-induced human hepatocytes (Li et al., 2021). Thus, we evaluated the effect of AMPK signal on oxidative stress induced by PO in hepatocytes. As we known, there is an antioxidant system in animals responsible for scavenging the ROS. Under normal physiological conditions, the production and removed of ROS are in dynamic equilibrium; once the decline of antioxidant capacity or ROS overproduction, which will cause the imbalance of antioxidant system and that induce oxidative stress in body (Islam, 2017). In the present study, AMPK inhibitor compound C pretreatment markedly aggravated the oxidative stress and the decrease of antioxidant capacity that triggered by PO in primary chicken hepatocytes. Conversely, pretreatment with AMPK activator AICAR significantly alleviated the PO-challenged oxidative stress and attenuated the decrease of antioxidant capacity induced by PO in primary chicken hepatocytes. These results suggested that AMPK acts beneficial relieving effect on the oxidative stress challenged by PO in chicken hepatocytes. In hepatocytes, the NRF-2 pathway plays a central role in controlling the occurrence of oxidative stress. As an important transcription regulator of antioxidant stress response, NRF-2 can bind to cytoplasmic chaperone KEAP1 that lead to the inhibition of its activity in physiological state (Solano-Urrusquieta et al., 2020); while, the NRF-2 dissociated from KEAP1 and transferred into the nucleus under the condition of oxidative stress, and then increased the antioxidant related factors (such as HO-1, NQO-1, and SOD) expression and improved antioxidant capacity (Saleh et al., 2019). However, excessive oxidative stress for a long time will block the NRF-2 transfer into the nucleus and then reduce the antioxidant capacity, which finally lead to a vicious circle of oxidative stress in the body. In the present study, we found that AMPK attenuated the oxidative stress triggered by PO mainly through activation of NRF-2 signaling pathway in primary chicken hepatocytes. As an upstream regulator of NRF-2, activated AMPK signal can induce the dissociation of NRF-2 from KEAP1, and then lead to NRF-2 into the nucleus and enhance the antioxidant factors expression level or activity in mammals (Su et al., 2021). Taken together, these data suggested that activated AMPK improves antioxidant capacity and then prevents oxidative stress induced by PO through activation of NRF-2 signaling pathway in primary chicken hepatocytes.

Many studies have reported that oxidative stress is closely related to inflammatory response (Reuter et al., 2010; Wang et al., 2021). Xing *et al* (Xing et al., 2020) reported that high-energy and low-protein diets can induce inflammatory response in ovaries of laying hens. In addition, mumerous studies have showed that the activation of NF-κB signaling pathway plays an important role in inflammatory response (Kawai and Akira, 2007; Zusso et al., 2019). In addition, our recent study reported that (-)-hydroxycitric acid prevents oleic acid-induced inflammatory response by activation of AMPK signal and which subsequently inhibit the activation of ROS-induced NF-κB signaling pathways (Li et al., 2020). In this study, we found that the upregulation of proinflammatory mediators level induced by PO-stimulated were significantly exacerbated in hepatocytes by pretreatment with AMPK inhibitor compound C, but that were alleviated in hepatocytes by pretreatment with AMPK activator AICAR. Accumulating evidences indicated that NF-κB signaling pathway was closely related to the release of proinflammatory mediators, and inhibition of NF-κB signaling pathway is an effective strategy in alleviating the inflammatory response (Oeckinghaus et al., 2011). Therefore, to further evaluate the anti-inflammatory effect of AMPK whether is associate with the inhibition of NF-κB signaling pathway, the related protein levels in NF-κB signaling pathway were detected in the current study. The results showed that the activation of NF-κB signaling pathway challenged by PO was markedly exacerbated in primary chicken hepatocytes pre-treated with compound C; however, the activation of NF-κB signaling pathway triggered by PO was obviously dispelled in PO-stimulated primary chicken hepatocytes pre-treated with AICAR. Therefore, our data indicated that activated AMPK attenuates inflammatory response induced by PO through inhibition of NF-κB signaling pathway in primary chicken hepatocytes.

**FIGURE 9:**
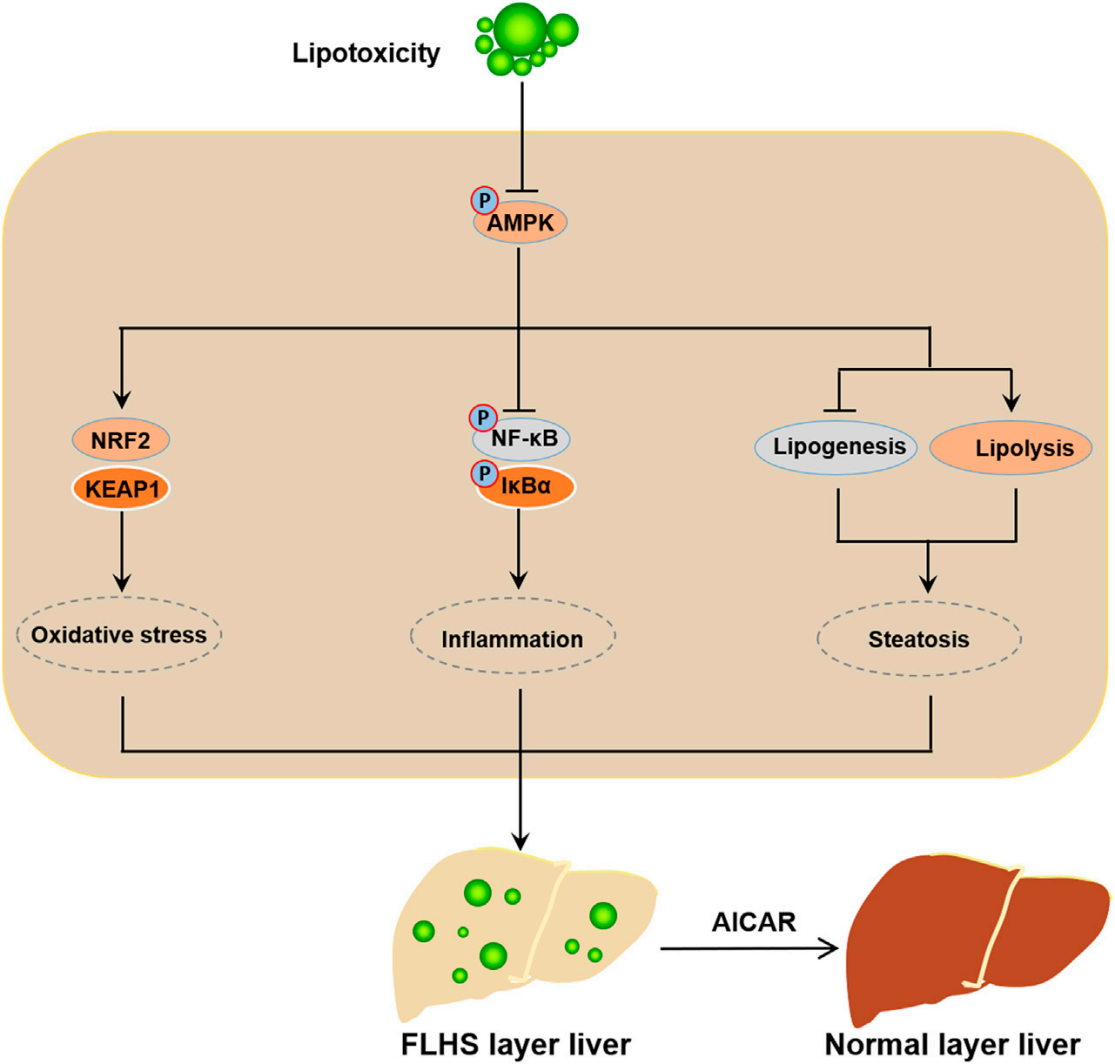
Schematic illustration of the mechanism of activated AMPK prevents the occurrence and development of FLHS. The mitigative effect of activated AMPK signal on the lipid metabolism disorder, oxidative stress and inflammatory response trigerred by PO-stimualtion is mainly achieved through the activation of NRF-2 signaling pathway and the inhibition of NF-κB signaling pathway in primary chicken hepatocytes; which implied that AMPK may be a potential target factor for the prevention of FLHS occurrence in laying hens.

In conclusion, our data demonstrated that activated AMPK presents an obvious relieving effect on the PO-stimulated lipid metabolism disorders, oxidative stress and inflammatory response; conversely, inhibition of AMPK exacerbates the PO-induced ipid metabolism disorders, oxidative stress and inflammatory response in primary chicken hepatocytes. Mechanistically, AMPK prevents lipid metabolism disorders, oxidative stress and inflammatory response triggered by PO mainly through the activation of NRF-2 signaling pathway and the inhibition of NF-κB signaling pathway in primary chicken hepatocytes (Figure 9). Collectively, these data suggested that AMPK may be an important target factor for the prevention of FLHS in laying hens.

## Data Availability

The original contributions presented in the study are included in the article/supplementary material, further inquiries can be directed to the corresponding author.

## Abbreviations

FLHS, fatty liver hemorrhagic syndrome; AMPK, AMP-activated protein kinase; PO, palmitic acid plus oleic acid; NRF-2, nuclear factor erythroid 2-related factor 2; KEAP1, kelch-like ECH-associated protein 1; MDA, malondialdehyde; T-AOC, total antioxidant capacity; SOD, superoxide dismutase; CAT, catalase; POD, peroxidase; ROS, reactive oxygen species; mtROS, mitochondrial reactive oxygen species; MMP, mitochondrial membrane potential; NQO1, NAD (P) H: quinone oxidoreductase 1; HO-1, heme oxygenase 1; TG, triglyceride; TC, total cholesterol; CD36, fatty acid translocase; SREBP-1c, sterol regulatory element binding protein 1c; ACCα, acetyl-CoA carboxylase α; FASN, fatty acid synthase; PPARα, peroxisome proliferator activated receptor α; CPT-1, carnitine palmitoyl transterase-1; IL-6, interleukin 6; TNF-α, tumor necrosis factor α.
